# Notch Signaling and T-Helper Cells in EAE/MS

**DOI:** 10.1155/2013/570731

**Published:** 2013-11-14

**Authors:** Ribal Bassil, William Orent, Wassim Elyaman

**Affiliations:** Center for Neurologic Diseases, Brigham and Women's Hospital, Harvard Medical School, Boston, MA 02115, USA

## Abstract

The Notch signaling pathway preservation across species hints to the indispensable role it plays during evolution. Over the last decade the science community has extensively studied the Notch signaling pathway, with Notch emerging as a key player in embryogenesis, tissue homeostasis, angiogenesis, and immunoregulation. Multiple sclerosis (MS) is an incurable yet treatable autoimmune chronic inflammatory disease of the central nervous system. The aim of this review is to provide a brief description of the Notch signaling pathway, and summarize the current literature implicating Notch in the pathogenesis of MS.

## 1. Introduction

The evolutionary conserved Notch signaling pathway is a crucial player in cell fate decision from embryogenesis to adult life and plays a key role in a broad range of cellular processes including activation, proliferation, differentiation, and apoptosis. Notch signaling orchestrates normal cell and tissue development and has been implicated in the pathogenesis of some of the most challenging medical problems facing our society. In this review, we are going to focus on the influence of this pathway on autoimmune diseases.

The canonical Notch signaling cascade is initiated when a Notch receptor engages a Notch ligand expressed on a neighboring cell. This triggers a series of enzymatic reactions leading to the release of the Notch receptor intracellular domain, which translocates to the nucleus and forms an active transcription complex regulating target genes expression [1–3]. 

Multiple sclerosis (MS) is a chronic, often disabling autoimmune inflammatory demyelinating disease of the central nervous system (CNS) affecting mostly the young adult population. Unknown environmental factors still under investigation are thought to trigger MS in genetically predisposed individuals. T-helper (Th) cells, so called for their ability to coordinate and fine-tune the immune response, initiate an attack against “self” antigens expressed mainly on oligodendrocytes (OLs) leading to chronic inflammation [4]. Notch signaling has been shown to regulate the development and function of both Th cells and OLs, with several groups reporting on the potential therapeutic implications of Notch pathway targeting in MS.

## 2. Notch Signaling

In 1914, John S. Dexter described a heritable “beaded” wing phenotype in the fruit fly *Drosophila melanogaster*. Twelve years later, Thomas H. Morgan published his work *The Theory of the Gene* in which he identified multiple mutant alleles resulting in this heritable “notched” wings phenotype. The gene was therefore appropriately called *Notch*. The Notch signaling pathway is now recognized as a cornerstone of cell-to-cell communication.

**Figure 1:**
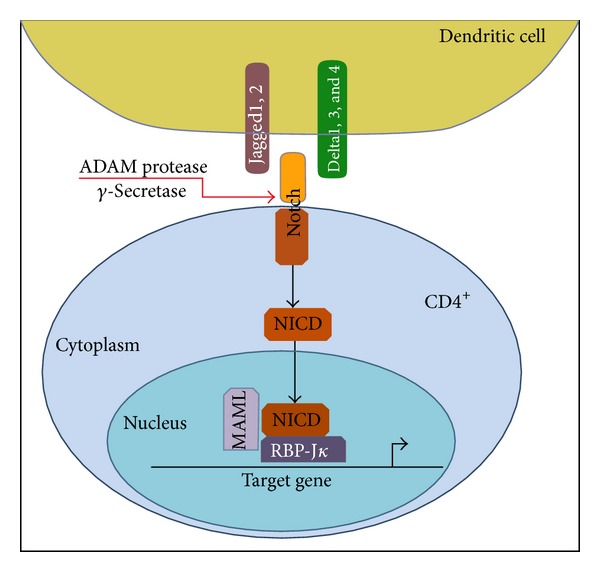
Schematic illustration of Notch signaling pathway. Binding of the extracellular part of Notch receptor to ligands of the Delta and Jagged families induces proteolytic cleavage of Notch, releasing the intracellular part of the protein (NICD). NICD is then translocated to the nucleus and binds to the nuclear transcription factor RBP-J*κ* inducing its conversion from a repressor into an activator to stimulate the transcription of Notch target genes.

In humans, the classic Notch signaling pathway consists of four heterodimeric transmembrane receptors (Notch 1, 2, 3, and 4) and their ligands (Delta-like 1, 3, and 4 and Jagged 1 and 2) [1]. The Notch receptor engagement by its ligand expressed on an adjacent cell is followed by two consecutive proteolytic reactions mediated by ADAM metalloproteases and the Presenilin family of *γ*-secretases. These enzymatic reactions lead to the cleavage of the receptor in its transcellular domain region, releasing the Notch intracellular domain (NICD) which then translocates to the nucleus. Once in the nucleus, NICD forms a transcriptional complex with the recombination signal binding protein for immunoglobulin kappa J region (RBP-J*κ*) and the coactivator mastermind-like (MAML) proteins, thus converting RBP-J*κ* from a transcriptional repressor to a transcriptional activator. The NICD/RBP-J*κ*/MAML complex then modulates the expression of their target genes [2, 3] (Figure 1).

## 3. T-Helper Cell Differentiation

Three signals are required for efficient T cell differentiation. The first is in the form of antigen presented by an antigen-presenting cell (APC), such as a dendritic cell (DC). The second signal comes in the form of costimulatory receptors on T cells engaging their cognate ligands on APCs. Small signaling protein molecules, that is, cytokines, provide the third signal [5]. Albeit an oversimplification, Notch signaling falls under the third signal category and fine-tunes the T cell response [6].

**Figure 2:**
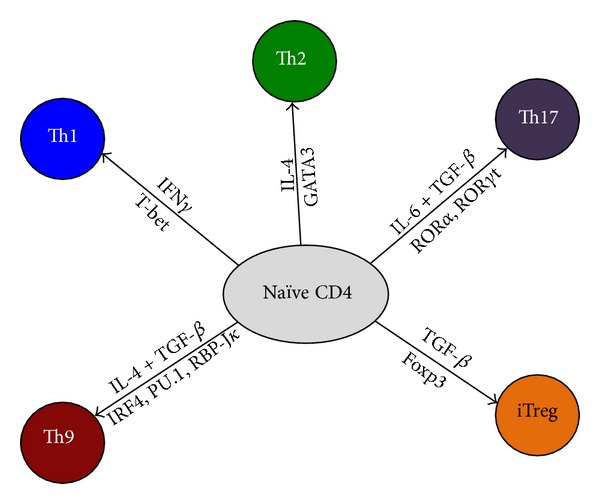
Schematic illustration of CD4^+^ T cell differentiation into effector or regulatory T cells. Depending on the cytokine milieu (shown above the arrows) present at the time of the initial engagement of their TCR, naive CD4^+^ T cells can differentiate into various subsets of T-helper cells (Th1, Th2, Th9, and Th17). However, in the presence of TGF-*β*1, naive T cells convert into foxp3-expressing induced Treg (iTreg) cells. For each T-helper cell differentiation program, specific transcription factors (shown below the arrows) have been identified as master regulators (T-bet, GATA3 and ROR*γ*t) for Th1, Th2, and Th17, respectively. IRF4, PU.1, and RBP-J*κ* transcription factors have been shown recently to contribute to the induction to Th9 cells.

To date, numerous T-helper cell subsets have been defined mainly based on the expression of master transcriptional regulators and cytokine production profiles (Figure 2) [7]. Antigen presentation in the presence of IL-12 induces the expression of T-bet and production of IFN-*γ*, therefore promoting naïve T cell polarization into the Th1 phenotype. IL-4 induces GATA3 expression and IL-4 production and is necessary for Th2 cell polarization. IL-6 and TGF-*β* induce ROR*γ*t expression and IL-17 production in Th17 cells. TGF-*β* is necessary for foxp3 expression and regulatory T cell (Treg) differentiation. The IL-9 producing Th (Th9) cells require both IL-4 and TGF-*β*, which induce IRF4 and PU.1 expression, respectively [7].

While Th cell subsets are necessary for providing immunity against infectious pathogens, their aberrant response is to blame in several medical problems such as autoimmune diseases, allergies, and malignancies. Therefore, a Th cell type could be either “good” or “bad” depending on the immunological context. Studies in humans as well as in animal models of MS suggest that Th1 and Th17 cells are mostly pathogenic, while Th2 and Treg cells are anti-inflammatory. The role of Th9 cells in autoimmune diseases is still controversial as they might be a plastic, nonterminally differentiated phenotype [8].

## 4. Delta-Like Ligands and Th Subsets

Several *in vitro* studies support a role for Delta-like ligands (Dll) in promoting Th1 cell differentiation [9–11]. Briefly, APCs expressing Dll promote Th1 while suppressing Th2 cell differentiation. Concurrently, exogenous stimuli that would enhance APCs polarizing potential of Th1 cells also increase the APCs expression of Dll [9]. RBP-J*κ* and NICD were reported to bind to the *Tbx21* and *Ifng* promoters, respectively, two hallmarks of Th1 cells [10, 11].

With regard to Th17 cells, Mukerjee et al. show that under Th17 polarizing conditions rDll4 treatment significantly enhances IL-17 production while *γ*-secretase inhibitor (GSI) mediated inhibition of Notch signaling abrogates it. Furthermore, RBP-J*κ* was found to bind to the *Il17* promoter and this was reduced in the presence of GSI [12].

Bassil et al. show that Dll4 mediated signaling inhibits TGF-*β*-induced Treg development as well as Janus kinase 3-induced STAT5 phosphorylation, a transcription factor known to play a key role in Foxp3 expression and maintenance [13]. The role of Dll4 in Treg development was further confirmed by Billiard et al. by showing that anti-Dll4 Ab treatment converts early T cell progenitors to immature tolerogenic DCs that promote Treg-cell expansion [14]. Adding another dimension to the picture, Hue et al. demonstrate that pretreatment with Notch ligands Dll4 and Jagged1 sensitizes CD4^+^CD25^−^ effector T cells to Treg-cell mediated suppression through increased TGF-*β*RII expression and Smad3 phosphorylation [15].

## 5. Jagged Ligands and Th Subsets

What applies to the Dll and Th1/Th2 cells is almost opposite to the findings seen with the Jagged ligands. APCs expressing Jagged ligands promote Th2 cells while suppressing Th1 cell differentiation. Concurrently, pathogens that enhance APCs polarizing potential for Th2 cells also increase the APCs expression of Jagged ligands [9]. Furthermore, Notch and RBP-J*κ* were found to bind the *Gata3* promoter and the HS5 site of the *IL4* enhancer, both critical genes in Th2 cell differentiation [9, 16, 17].

Jagged ligands are thought to enhance the development and function of regulatory T cells. In a human *in vitro* study, Vigouroux et al. report on the induction of an antigen specific IL-10 producing regulatory T cell population (Tr1) following stimulation by Jagged1 transduced B cells [18]. Kared et al. show that a population of hematopoietic progenitor cells (HPCs) highly expressing Jagged2 ligand activated Notch3 signaling in Treg cells enhancing their expansion and suppressive function. This signaling mechanism required cell-to-cell interaction and was inhibited by GSI [19].

Asano et al. have demonstrated that Treg suppressor cells express Jagged1 while the responder cells (CD4^+^CD25^−^) express Notch1. Anti-Notch1 and to a lesser extent anti-Jagged1 Abs inhibited the suppressive function of Treg cells. Furthermore, they show that Jagged1-mediated Notch1 activation enhances TGF-*β*-induced Smad3 transcription and translocation to the nucleus, a key component of TGF-*β* mediated signaling [20].

With regard to Th9 cells, Elyaman et al. have found Notch1 and Notch2 conditional ablation to significantly reduce IL-9 production. In fact, Jagged2 mediated Notch signaling promotes RBP-J*κ*/NICD1/Smad3 transcriptional complex formation and binding and transactivation of the *Il9* promoter [21].

## 6. Notch Intracellular Domain and Noncanonical Signaling in Th Subsets

In addition to the data that has been generated involving the Delta-like and Jagged ligands, a plurality of data has been generated without regard to ligand to show Notch involvement in T-helper subset differentiation, and more work will need to be done to fully elucidate the specific ligand pathway.

RBP-J*κ* and NICD have been shown to bind the Gata3 promoter, without specific ligand activation [16, 17]. Similar results have been shown for the Tbx21 and Ifng promoters as well [8, 9]. Thus the specific ligand pathway of many aspects of Notch signaling remains to be determined despite consistent results showing involvement in Th development.

Another topic of active research is the role of noncanonical Notch signaling in Th differentiation. Perumalsamy et al. found that NICD in the plasma membrane, rather than the nucleus, was associated with improved survival of Tregs [22]. Additionally Auderset et al. showed Notch signaling independent of RBP-J*κ* to be important for Th1 development during parasitic infections [23]. The increasing body of evidence points to a significant role for noncanonical Notch signaling in the differentiation and proliferation of Th subsets (see Table 1), and this will likely be an active area of research in the future.

## 7. Notch and Oligodendrocytes

Oligodendrocyte (OL) projections provide neurons with a protective and insulating myelin sheath, which optimizes nerve conduction speeds. The autoimmune response targeting this myelin sheath results in slowing nerve conduction velocities and is responsible for the neurological deficits in MS. Therefore, immunoregulatory approaches targeting oligodendrocyte progenitor cell (OPC) proliferation and differentiation would be invaluable. It is worth noting that several groups have demonstrated that the timing of Notch signaling differentially regulates OPC development, with Dll1- and Jagged1-mediated signaling inhibiting OPC maturation while enhancing their expansion [24–26].

## 8. Notch and Animal Models of MS

Experimental autoimmune encephalomyelitis (EAE), the most widely used model for MS [27, 28], is induced by active immunization of mice with myelin antigens emulsified in adjuvant [29]. Alternatively, EAE can be induced by passive transfer of activated myelin-specific cellular clones or cell lines [30]. Theiler's murine encephalomyelitis virus-(TMEV-) induced demyelinating disease (TMEV-IDD), another popular model for MS, is induced by intracerebral injection with TMEV resulting in CNS inflammation [28]. 

Minter et al. nonspecifically inhibited Notch signaling by oral or intraperitoneal administration of GSI in the PLP/SJL EAE model. This resulted in a significant decrease in clinical disease and Th1 associated cytokines reduction [10]. Keerthivasan et al. followed up on this work by showing that Notch plays a role in Th17 differentiation and GSI in the PLP/SJL EAE model reduces IL-17 production [31].

Jurynczyk et al. provided compelling evidence that Notch3 may play a significant role in EAE when they showed that, by using GSI against specific Notch3 and not Notch1, there is a significant decrease in clinical disease score as well as Th1 and Th17 cytokines using the PLP/SJL EAE model [32].

Among all Notch ligands, the role of Dll4 in animal models of MS has been the most studied role. In 2010, Takeichi et al. showed that Dll4 expression is significantly upregulated on DCs in the TMEV-IDD model. Dll4 blockade significantly ameliorated the clinical course of the disease, which was attributed to a decrease in mononuclear cell infiltration of the target tissues and reduction in IFN-*γ* and IL-17 production [37].

In 2011, in concordance with the TMEV-IDD study, Reynolds et al. described an increase in Dll4 expression on APCs in the PLP/SJL EAE model, with Dll4 blockade alleviating clinical disease and decreasing IFN-*γ* and IL-17 producing CD4^+^ T cells frequency and leukocyte infiltration of the CNS, while having no effect on the Foxp3 mRNA expression levels. Reynolds et al. attribute the effects observed with Dll4 blockade to a downregulation of the chemokine receptors CCR2 and CCR6 expression on CD4^+^ T cells, leading to their differential migration and accumulation in the CNS [38]. Also in 2011 and in agreement with the previous studies, Bassil et al. showed that Dll4 blockade in the MOG/B6 EAE model alleviates the clinical EAE severity and shifts the immune balance from a Th1/Th17 mediated response toward a Th2/Treg mediated response. In this study, the effects were mainly attributed to the role Dll4 plays in regulating Treg development, with Treg depletion prior to EAE induction abrogating the anti-Dll4 mAb protective effect [13].

Dll1 contribution to the EAE model has been described by Elyaman et al. in 2007, showing DC upregulation of Dll1 expression during the induction phase of the disease. Dll1 blockade reduced the disease severity and CD4^+^IFN-*γ* ^+^ cell frequency, while Dll1 ligation had the opposite effect. Modulation of the Dll1 mediated signaling had no effect on CD4^+^Foxp3^+^ cell frequencies [35]. Tsugane et al. reported on Dll1 blockade in the TMEV-IDD model in 2012. A decrease in IFN-*γ*, IL-4, and IL-10 producing CD4^+^ T cells and an increase in IL-17 producing CD4^+^T cells were observed in the spinal cords of treated mice. This resulted in a significant suppression of the disease both clinically and histologically [36].

The role of the Jagged ligands in animal models of MS has not been studied as much as their Dll counterparts. Our group has shown that the administration of anti-Jagged1 mAb exacerbated EAE clinical disease and was associated with a decrease in IL-10-producing CD4^+^ T cells in the CNS. In contrast, the administration of Jagged1-Fc protected the mice from disease and increased the frequency of IL-10-producing CD4^+^ T cells [35]. Using a human Jagged1 agonist peptide, Palacios et al. have also concluded that Jagged1 signaling ameliorates EAE course, which was associated with an increase in CD25^+^Foxp3^+^ T cell frequency [39]. In a recent study, Elyaman et al. reported that the timing of Jagged2 mediated signaling differentially regulates EAE. In that report, we show that Notch signaling is required for optimal IL-9 production. Jagged2 signaling molecule administration before antigen immunization promotes IL-9-mediated Treg-cell expansion and suppresses EAE, while Jagged2 signaling molecule administration concurrent with immunization worsens EAE, with IL-9 favoring Th17 cell expansion in this inflammatory milieu [21]. The role of Notch signaling in animal models of MS is summarized in Table 2.

Notch signaling has been investigated in other models of immune mediated diseases and the data complements the findings in the EAE system. Not surprisingly, the effect on the clinical disease was largely dependent on the immunological context. The data is summarized in Table 3.

## 9. Notch and MS

Despite the overwhelming evidence supporting the role of Notch signaling in Th cell development and in regulating the outcome in animal models of MS, studies in the human system remain scarce and mostly point to Jagged1 or were ligand independent.

Zhang et al. studied chronic active MS lesions and concluded that the expression of Jagged1 in remyelinated MS lesions is nonsignificant. On the other hand, in active MS lesions lacking remyelination, Jagged1 is highly expressed by hypertrophic astrocytes, with Notch1 being preferentially expressed in nondifferentiated OLs [26]. In a study of chronic silent MS lesions, Nakahara et al. observed a high level of activation of Notch1 through the noncanonical Notch signaling pathway, while the classic Notch signaling pathway is inhibited [48].

An analysis of gene networks regulating T cell activation in MS patients by Palacios et al. has concluded that *Jagged1* is consistently modified in the disease state making it a potential therapeutic target in MS [39]. However, the strongest inculpating evidence emerged in 2006 when a meta-analysis of the Genetic Analysis of Multiple Sclerosis in EuropeanS (GAMES) project involving 13,896 individuals identified *Jagged1* as a susceptibility gene for MS [49].

These observations taken together with the data from *in vitro* studies further highlight the key role of the Notch signaling pathway in regulating the immune balance in MS.

## 10. Concluding Remarks

The scientific community has provided overwhelming evidence implicating the Notch signaling pathway in the pathogenesis of autoimmune diseases including MS. Notch-mediated signaling emerges as a key regulator of the development of Th cell subsets promoting autoimmunity, as well as other Th subsets playing an anti-inflammatory role [4, 10, 13, 21, 35]. This dichotomy has also been demonstrated in OPCs where the nature and timing of Notch signaling could either enhance or inhibit OPC maturation and expansion [25, 26]. Therefore, Notch signaling regulates the development and function of pathogenic cells as well as cells with regenerative and anti-inflammatory properties. This makes Notch signaling targeted immunotherapy extremely promising yet problematic for the same reason. To complicate the picture, while it seems likely that Th subsets are a valid target for Notch immunotherapy, APCs and other myeloid cells clearly play a role in EAE and should not be excluded as potential cell-specific targets.

The obvious challenges arise from the difficulties in delivering the right immunomodulatory signal to the right target cell at the right time. To further complicate the picture, Notch receptors and ligands are ubiquitously expressed making the nonselective approach less than ideal. We believe that the current literature supports and encourages a Notch signaling targeted immunotherapy even in a noncell-specific targeting system through the use of signaling pathway inhibitors such as GSI or the use of mAbs and signaling molecules. However, harnessing the immense therapeutic potential of the Notch signaling pathway modulation lies in taking advantage of future advances and breakthroughs in cell-specific targeted drug delivery systems.

## Figures and Tables

**Table 1:** Notch and Th subsets.

Ligand/pathway	Method	Results	References
Dll	BMDC LPS stimulation	**↑**Dll4 mRNA	Amsen et al., 2004 [9]
Dll	Dll1 expressing APC/CD4^+^ T cells coculture	**↑**IFN-g	
Dll	CD8^−^ DCs LPS stimulation	**↑**Dll4 expression	Skokos and Nussenzweig, 2007 [33]
Dll	Dll4-mFc CD4^+^ T cell treatment	**↑**IFN-g	
Dll	DCs TLR2/TLR9 ligation	**↑**DCs Dll expression, **↑**T-bet, **↑**IFN-g, and **↓**IL-4 by CD4^+^ T cell	Sun et al., 2008 [34]
Dll	CD4^+^ T cell recDll4 treatment	**↑**RORc activation, **↑**IL-17	Mukherjee et al., 2009 [12]
Dll	CD4^+^ T cell recDll4 treatment	**↓**phospho-Jak3, **↓**phospho-Stat5, and **↓**Foxp3	Bassil et al., 2011 [13]
Dll	CD4^+^ CD25^−^ cells Dll4 and Jagged1 pretreatment	**↑**TGF-*β*RII and phospho-Smad3	Hue et al., 2012 [15]
Jagged	Jagged1 transduction of human APCs	Induction of IL-10 producing Tr1 cells	Vigouroux et al., 2003 [18]
Jagged	HPCs expressing Jagged2	**↑**Treg expansion and function	Kared et al., 2006 [19]
Jagged	Notch1 or Jagged1 blockade	**↓**Treg function	Asano et al., 2008 [20]
Jagged	BMDC LPS stimulation	**↑**Jagged1 mRNA	Amsen et al., 2004 [9]
Jagged	Jagged1 expressing APCs/CD4^+^ T cells coculture	**↑**IL-4, **↑**IL-5	
NICD	NICD forced expression in CD4^+^ T cells	NICD regulates *IL4* transcription	
NICD	RBP-J*κ*/NICD1/Smad3 forced expression in CD4^+^ T cells	RBP-J*κ*/NICD1/Smad3 complex binds and transactivates *Il9* promoter	Elyaman et al., 2012 [21]
NICD	Cell line transduction	RBP-J*κ* binds *Tbx21* promoter	Minter et al., 2005 [10]
NICD	Splenocytes aCD3/aCD28 stimulation	NICD binds *Ifng* promoter	Shin et al., 2006 [11]
NICD	NICD forced expression in CD4^+^ T cells	NICD binds the *Gata3* promoter	Fang et al., 2007 [17]
NICD	Notch1 blockade in Th17 cells	**↓**Th17 associated cytokines	Keerthivasan et al., 2011 [31]
NICD	Cell line transfection	RBP-J*κ* binds the *Gata3* promoter	Amsen et al., 2007 [16]
Noncanonical	*In vivo* notch ablation in CD4^+^ cells	Notch1 and Notch2 redundantly essential for Th1 development	Auderset et al., 2012 [23]
Noncanonical	Mutant NICD in Notch1 KO Tregs	NICD targeting plasma membrane improves Treg survival	Perumalsamy et al., 2012 [22]

**Table 2:** Notch and animal models of MS.

MS animal model	Method	Results	References
EAE (PLP/SJL)	GSI	**↓**Disease, **↓**Th1	Minter et al., 2005 [10]
EAE (PLP/SJL)	Anti-Notch3	**↓**Disease, **↓**Th1, and **↓**Th17	Jurynczyk et al., 2008 [32]
EAE (PLP/SJL)	GSI	**↓**Disease, **↓**Th17	Keerthivasan et al., 2011 [31]
EAE (MOG/B6)	Anti-Dll1	**↓**Disease, **↓**Th1	Elyaman et al., 2007 [35]
TMEV-IDD	Anti-Dll1	**↓**Disease, **↓**IFN-*γ*, and **↓**IL-4	Tsugane et al., 2012 [36]
TMEV-IDD	Anti-Dll4	**↓**Disease, **↓**IFN-*γ*, and **↓**IL-17	Takeichi et al., 2010 [37]
EAE (PLP/SJL)	Anti-Dll4	**↓**Disease, **↓**Th1, and **↓**Th17	Reynolds et al., 2011 [38]
EAE (MOG/B6)	Anti-Dll4	**↓**Disease, **↓**Th1, **↓**Th17, **↑**Th2, and **↑**Treg	Bassil et al., 2011 [13]
EAE (MOG/B6)	Anti-Jagged1	**↑**Disease, **↓**IL-10	Elyaman et al., 2007 [35]
EAE (MOG/B6)	Jagged1 peptide	**↓**Disease, **↓**IFN-*γ*, and **↑**IL-4	Palacios et al., 2007 [39]
EAE (MOG/B6)	Anti-Jagged2 signaling molecules prior to immunization	**↓**Disease, **↑**Treg	Elyaman et al., 2012 [21]
	Anti-Jagged2 signaling molecules at time of immunization	**↑**Disease, **↑**IL-17	

**Table 3:** Notch and animal models of immune mediated diseases.

Animal model	Method	Results	References
Allergic conjunctivitis	Anti-Dll4	↑Disease, **↑**Th2	Fukushima et al., 2008 [40]
Allergic asthma	Anti-Dll4	**↑**Disease, **↓**Treg function	Huang et al., 2009 [41]
Allergic airway response	Anti-Dll4	**↑**Disease, **↑**Th2	Jang et al., 2010 [42]
Autoimmune uveoretinitis	Anti-Dll4	**↑**Disease, ↓Th17	Ishida et al., 2011 [43]
T1D	Anti-Dll4	↓Disease, **↑**Treg	Billiard et al., 2012 [14]
Graft versus host disease	Anti-Dll4	**↑**Survival, **↓**Th1, and **↓**Th17	Mochizuki et al., 2013 [44]
Allogeneic cardiac transplant	Anti-Dll1	**↑**Survival, **↓**Th1, and **↓**cytotoxic T cell	Riella et al., 2011 [45]
Airway hyperresponsiveness	Jagged1-Fc	**↑**Disease, **↑**Th2	Okamoto et al., 2009 [46]
Murine cardiac transplant	Anti-Jagged2 signaling Ab	**↓**Survival, ↑IL-2, and ↑IL-6	Riella et al., 2013 [47]
